# 

**Published:** 1882-01-20

**Authors:** 


Entered at the Post-Office at Atlanta as second-class matter.

VOL. XII. JANUARY 20, 1882. NO. 1.

THE

### SOUTHERN MEDICAL RECORD:

A MONTHLY JOURNAL OF PRACTICAL MEDICINE.

Terms: $2 00 per Annum, in Advance; 4 Copies $6.00; Single Copies 20 Cents.

*"QUICQUID PRÆCIPIES ESTO BREVIS."*

| | | | |
|---|---|---|---|
| William R. Warner & Co. | 1 | Mellin's Food, | 14 |
| Celerina | 2 | N. Y. Pharmacal Association | 15 |
| Petrolina | 3 | Geo. J. Howard & Bro. | 16 |
| J. L. Berg & Co. | 4-5 | Kidder & Laird, | (Inside front cover) |
| Powell's Combined Beef, etc. | 6 | J. Bradfield, | (colored leaves) |
| A. A. Mellier | 7 | Allen & Masser, | (colored leaves) |
| Sharp & Dohme | 8 | Reed & Carnrick | (inside back cover.) |
| Johnston's Fluid Beef | 9 | Parke, Davis & Co. | (outside back cover.) |
| Thomas F. Goode | 10 | Parke, Davis & Co. | (colored leaves.) |
| O. C. St. Clair | 11 | Scott's Emulsion | (colored leaves.) |
| Dundas, Dick & Co. | 12 | Listerine | (colored leaves.) |
| Bedford Springs | 13 | Kidder & Laird | (colored leaves.) |

Address R. C. WORD, M.D., Managing Editor, Atlanta, Ga.

